# Photocycle of point defects in highly- and weakly-germanium doped silica revealed by transient absorption measurements with femtosecond tunable pump

**DOI:** 10.1038/s41598-022-13156-7

**Published:** 2022-06-02

**Authors:** V. De Michele, A. Sciortino, M. Bouet, G. Bouwmans, S. Agnello, F. Messina, M. Cannas, A. Boukenter, E. Marin, S. Girard, Y. Ouerdane

**Affiliations:** 1 UJM, CNRS, IOGS, Laboratoire H. Curien, UMRCNRS 5516, Univ Lyon, 42000 Saint-Étienne, France; 2 Department of Physics and Chemistry “Emilio Segrè”, University of Palermo, Via Archirafi 36, 90123 Palermo, Italy; 3 Univ. Lille, CNRS, UMR 8523 - PhLAM - Physique des Lasers Atomes et Molécules, F-59000 Lille, France; 4 ATeN Center, University of Palermo, Viale delle Scienze, Edificio 18, 90128 Palermo, Italy

**Keywords:** Optical spectroscopy, Condensed-matter physics, Ultrafast photonics

## Abstract

We report pump-probe transient absorption measurements addressing the photocycle of the Germanium lone pair center (GLPC) point defect with an unprecedented time resolution. The GLPC is a model point defect with a simple and well-understood electronic structure, highly relevant for several applications. Therefore, a full explanation of its photocycle is fundamental to understand the relaxation mechanisms of such molecular-like systems in solid state. The experiment, carried out exciting the sample resonantly with the ultraviolet (UV) GLPC absorption band peaked at 5.1 eV, gave us the possibility to follow the defect excitation-relaxation dynamics from the femto-picosecond to the nanosecond timescale in the UV–visible range. Moreover, the transient absorption signal was studied as a function of the excitation photon energy and comparative experiments were conducted on highly- and weakly-germanium doped silica glasses. The results offer a comprehensive picture of the relaxation dynamics of GLPC and allow observing the interplay between electronic transitions localized on the defect and those related to bandgap transitions, providing a clear evidence that the role of dopant high concentration is not negligible in the earliest dynamics.

## Introduction

Amorphous SiO_2_ (silica) is a wide bandgap material that has been extensively studied in recent decades^1^, enabling the development of high-impact optical technology devices with applications in several fields: telecommunications, optics, sensors, diagnostics and many others^1–7^. Being an archetypal transparent solid, it has also contributed to improve our fundamental knowledge on the dynamics of point defects embedded in solid matrices^1,2,8,9^, which play a cardinal role on the optical responses of wide bandgap materials. Point defects can be pictured as deformations in the matrix, with dimensions close to the one of the silica structural unit. They are able of absorbing light from the near-infrared to the vacuum UV (VUV), degrading the transmission properties of transparent solids^1,3,8^.

In the present work, we focus our investigation on the germanium (Ge) doped silica^10,11^: a crucial issue since the incorporation of Ge in SiO_2_ leads to structural changes of the glass, with variation of its refractive index^12^. Nowadays, this possibility is exploited in bulk glasses or in other silica-based devices such as optical fibers^3,13,14^, enabling the control of their optical properties. Indeed, varying Ge-content in silica-based materials provides routes to optimize the different technological applications, enhancing the glass photosensitivity^15–17^ and/or inducing the breakdown of the inversion symmetry in the material, allowing non-linear optical phenomena such as the second harmonic generation^18^. From the structural point of view, Ge and Si atoms are isoelectronic^19^, resulting in the substitutional bonding of Ge at silicon sites, bonding to four O atoms, or in the formation of different Ge point defects, such as the under-coordinated germanium oxygen deficient centers (GeODC)^1,3,10,19,20^. Among those defects, here we report the ultrafast transient absorption response of the Germanium Lone Pair Center (GLPC), also known as the twofold-coordinated Ge^3,21,22^. This diamagnetic defect is composed of an under coordinated germanium atom bonded to two oxygen atoms and characterized by the presence of an electron lone pair, described as = Ge:, with a C_2v_ local symmetry. The GLPC optical activity has been long investigated, with well-known features^3,10,21–23^: (1) a UV absorption band peaked at 5.1 eV with a full width at half maximum (FWHM) of ~ 0.46 eV, with oscillator strength of ~ 0.12; (2) another transition in the vacuum UV, centered around ~ 7.6 eV, with an oscillator strength of the same order than the previous band. Upon excitation of those bands, two photoluminescent (PL) bands are observed: one peaked at 4.2 eV with a FWHM ~ 0.44 eV and a temperature dependent lifetime, τ ~ 1 ÷ 2 ns (at room temperature), while a second emission band peaked at 3.1 eV with a FWHM ~ 0.42 eV and a lifetime τ ~ 100 µs (forbidden transition). Despite its rich optical activity, the GLPC is a model point defect with a well-understood molecular-like electronic structure, enabling the investigation of the excitation-relaxation mechanisms which characterize such basic molecular like systems. Indeed, the two bands absorbing at ~ 5.1 eV and ~ 7.6 eV are related to the transitions involving the two S_1_ and S_2_ singlet states from the ground single state S_0_, while the electronic level absorbing at 3.8 eV is a T_1_ triplet excited level. The emission centered at 4.2 eV is associated with the S_1_ → S_0_ transition, inverse to the S_0_ → S_1_ absorption, and it is also excited by the S_0_ → S_2_ through an S_2_ → S_1_ internal conversion; its lifetime, of the order of the nanoseconds, is consistent with a singlet–singlet allowed transition. The population of the triplet state T_1_, being associated to a forbidden transition, is mostly built through an S_1_ → T_1_ intersystem crossing (ISC) with an associated relaxation time of ~ 1 ns. Despite the extensive knowledge on the GLPC’s electronic structure, the relaxation mechanisms upon UV excitation are still questionable, revealing the importance to apply innovative experimental and theoretical approaches. In particular, the contribution of non-radiative S_1_ → S_0_ depopulation versus the radiative one and the ISC at triplet level is still debated, as well as the nature of the ISC process itself, characterized by a non-Arrhenius behavior as a function of the temperature^24^. Understanding the fundamental role of triplet excited states in the dynamics of molecular like systems and their population/depopulation pathways through ISC and radiative/non-radiative relaxation, respectively, is a keystone to elucidate molecular photo-reactivity processes. In this context, the GLPC is one of the best candidates to address this challenge.

In this framework, our experiments will be focused on the investigation of the GLPC fast excitation/relaxation dynamics, taking advantages from the experimental approach already proposed in^25^, upon UV excitation around the absorption band centered at 5.1 eV and in linear absorption conditions. Moreover, based on the advantage that different germanium contents correspond to different energy bandgaps in the material, we carried out comparative transient absorption (TA) experiments^26^ in samples with high- and weak-Ge doping levels, acquiring new information about the influence of the energy landscape on the GLPC electronic levels and the relative relaxation channels. The experiment provides us enough information not only to propose a revised model for the GLPC photocycle, highlighting new features in a time window from hundreds of fs to the ns scale, but also on the unexpected role of the dopant concentration and delocalized bandgap electronic states on the short defect dynamics. The present approach demonstrates how ultrafast tunable pump-probe measurements represent the state of the art for investigating defect excitation/relaxation channels and dynamics.

## Methods

### Ge-doped samples

Two different Ge-doped silica samples were investigated to study the GLPC excitation/relaxation dynamics: (1) a silica based preform having an inner core doped with 6% of Ge (in the present paper the concentrations are expressed in wt%) while its cladding is made of pure silica material, with a size of 4 × 4 × 0.67 mm^3^; (2) 0.1% wt uniformly Ge-doped sample, size of 3 × 3 × 0.92 mm^3^. The 6% Ge-doped sample was obtained by MCVD (Modified Chemical Vapor Deposition) technique^27^, which is well-established method to manufacture optical fiber preforms. In this particular case, the pure silica glass tube (F300, Heraeus) was used as substrate and several layers of GeO_2_ doped silica were deposited inside the tube in controlled temperature, gas flow and precursor concentration. Then, tube was collapsed into solid rod and the external part was etched by the hydrofluoric acid in order to approach the central part. The 0.1% sample is synthetized with the sol–gel method^11,28^ starting from a mixture of two alkoxides. The silicon tetraethoxide (TEOS) solution, precursor of the amorphous silica matrix, was mixed with a solution of germanium tetraethoxide (TEOG), necessary for the doping. The pure silica sol, was obtained by adding the TEOS to an aqueous solution of HCl, using the ethanol as co-solvent. The solution was stirred to obtain a sol containing principally hydrolyzed TEOS, since the ethanol is removed by distillation. At this step, the TEOG was added very slowly to the silica sol under stirring. The final sol was poured in the molds and gelation occurred. For 24 h the gel was dipped in ethyl acetate, then it was autoclaved for 4 h at a temperature of 280 °C and a pressure P of 60 atm. The resulted aerogel was densified with heating up to 1150 °C with a rate of 20 °C/h and held for 24 h. During the heating, oxygen atmosphere was kept until 700 °C and then under vacuum. The investigated samples exhibit an intense absorption band around 245 nm (5.1 eV), footprint of the GLPC presence^3,11,21,22^. In addition, the Ge-content influences the structure of the silica, as observed through Raman measurements by the reducing low-membered rings of pure silica^29^, and leads to the reduction of the effective energy bandgap, as established by optical characterization of doped samples^15,29,30^.

### TA experimental setup

Pump/probe measurements were performed with a Yb:KGW (ytterbium-doped with potassium, gadolinium and tungstate fiber laser) fs laser, characterized by ~ 200 fs pulse duration and centered at 1030 nm (PHAROS: Light Conversion). A simplified picture of the experimental setup is displayed in Fig. S1, while a more detailed description is reported in^25^. The tunable excitation wavelength is generated through an OPA (optical parametric amplifier), from Light Conversion, delivering pulses at a repetition rate of 60 kHz. Samples were excited with femtosecond pulses in the UV range, at fixed energy per pulse of ~ 50 nJ/pulse, from 320 nm (3.9 eV) to 230 nm (5.4 eV), following the transient absorption resonantly with the 5.1 eV absorption band. The pump and the probe spots dimensions focalized into the samples have a diameter of ~ 100 µm and ~ 90 µm, respectively. The optical delay line allows us to control the delay between the two beams up to a maximum of ~ 7 ns. The transient absorption response, chopped at 75 Hz, was probed in the UV/visible (370 ÷ 650 nm) spectral range. The transmitted probe light is acquired using an imaging spectrograph (Kymera 193i, OXFORD instruments) equipped with a NMOS linear image sensor (HAMAMATSU S8380-256Q). Every spectrum is the average of 4000 spectra performed in the whole investigated window under these experimental conditions.

## Experimental results

**Figure 1:**
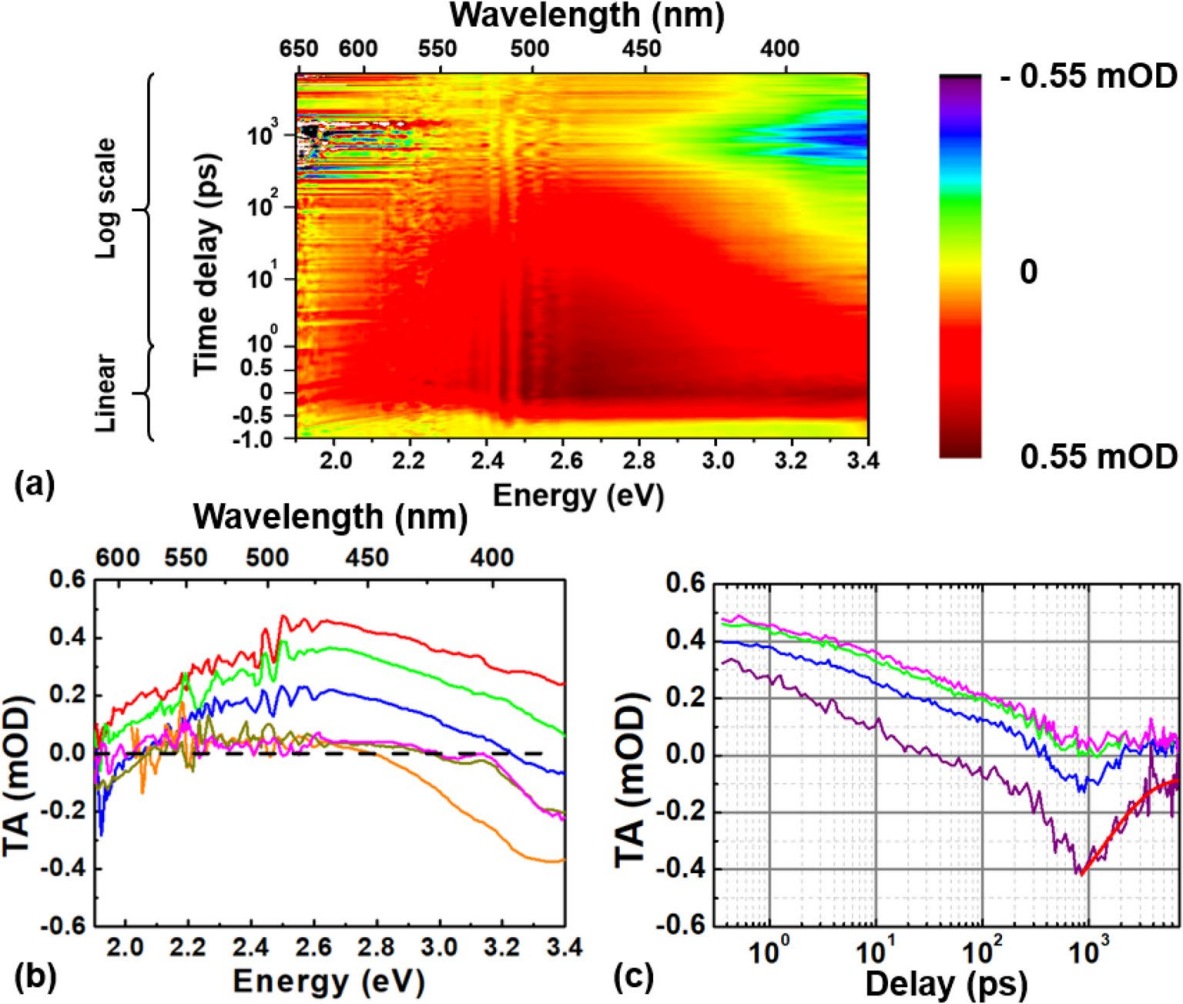
(**a**) Two-dimensional time-probing energy plot of the TA signal measured in the 6% Ge-doped preform sample after photo-excitation with 200 fs laser pulses at 5.1 eV and 50 nJ/pulse. (**b**) TA spectra recorded at different pump-probe delays: 1 ps in red, 10 ps in green, 100 ps in blue, 1 ns in orange, 3 ns in purple and 6 ns in magenta. The black dashed line is a guide to the eyes for the zero. (**c**) TA kinetics at different probing energies: in purple at 3.35 eV, in blue at 2.95 eV, in green at 2.75 eV and in magenta at 2.64 eV. The red line represents the decay time fitting with the best fit parameter τ = 1.46 ns of the negative component observed at 3.35 eV.

The TA response of the 6% Ge-doped sample, as a function of the pump-probe delay and the probing energy, is reported in Fig. 1a. The 2D color map is composed of a broad band with a peak located around 2.7 eV, that disappears within ~ 1 ns isolating a negative component at high energies in the nanosecond timescale. These observations are clearer from Fig. 1b, which displays the TA spectra recorded at different time delays. The broad main band, which covers the whole investigated spectral range from ~ 3.4 to ~ 1.9 eV, can be attributed to an excited state absorption (ESA) between two electronic levels, as expected from the S_1_ → S_2_ transition^21,22^. However, it should also be considered that the high germanium content decreases the energy bandgap as observed in previous investigations^15,29–31^. In a 5% doped sample the energy bandgap between the valence (VB) and the conduction bands (CB) is around ~ 7.1 eV. Considering this value as the maximum possible energy bandgap in the 6% Ge-doped specimen, the S_2_ electronic level is most likely degenerated with the CB, and becomes mixed with it. Then, the main band is consistent with a S_1_ → S_2_/CB transition, thus explaining the continuous wide observed ESA.

Moreover, it is possible to detect the rise of a negative component, reaching the minimum around ~ 1 ns (the orange curve in Fig. 1b) after which it decreases (see the comparison between the orange and the magenta curves in Fig. 1b at 1 ns and 6 ns, respectively). This provides evidence that the whole spectrum does not follow the same decay, the post excitation relaxation strongly depends on the probing energy.

Figure 1c shows TA kinetics recorded at different probing energies: 3.35 eV, 2.95 eV, 2.75 eV and at 2.64 eV. The comparison highlights that the UV component (3.35 eV) assumes negative values around ~ 10^2^ ps and reaches its minimum at ~ 10^3^ ps. Since the GLPC has no absorption band in the investigated probing range, it is reasonable to attribute such negative signal to a stimulated emission (SE) and, specifically, to the emission from the S_1_ to the S_0_ state. Based on the spectra at different pump probe delays (Fig. 1b), it seems that the SE starts to be relevant after ~ 10 ps for the highest probing energy. In fact, considering that the PL at 3.1 eV from the T_1_ → S_0_ transition has a very low radiative rate (decay time τ = 110 μs), the only SE, that could be observed, stems from the tail of the PL band centered at 4.2 eV (decaying in a few ns).

The negative component at 3.35 eV (Fig. 1c) ultimately decays over a time scale of a few nanoseconds. We performed a fitting routine considering an exponential decay (with a negative amplitude) to determine its decay time τ. As reported in Fig. 1c, the best fit parameter leads to a time decay of τ ~ 1.46 ns, which strongly agrees with the characteristic decay time of the GLPC luminescence at 4.2 eV measured at room temperature (~ 293 K)^15,19,21^. Then, this negative feature can be conclusively attributed to the tail of the PL band centered at 4.2 eV.

**Figure 2:**
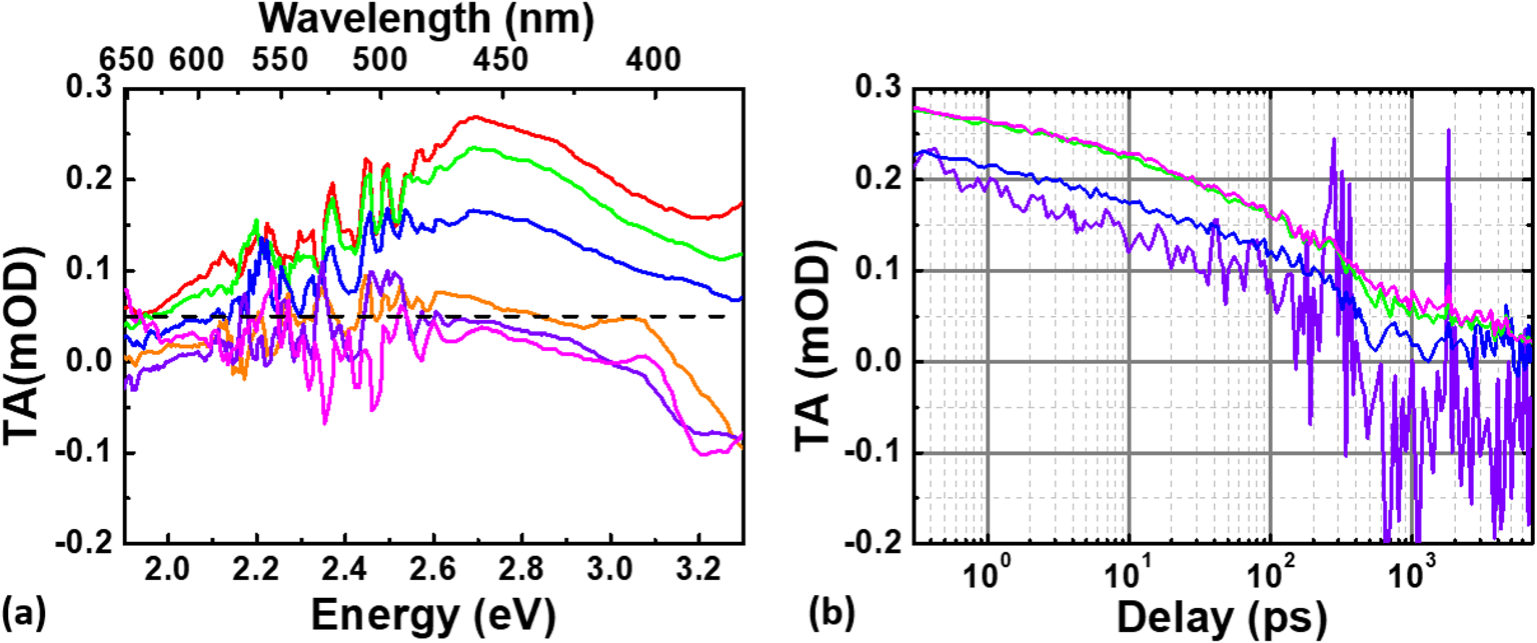
(**a**) TA spectra at different pump-probe delays measured in the 0.1% Ge-doped sample upon UV excitation at 5.1 eV and 50 nJ/pulse: 1 ps in red, 10 ps in green, 100 ps in blue, 1 ns in orange, 3 ns in purple and 5 ns in magenta. (**b**) TA kinetics at different probing energies: in purple at 3.30 eV, in blue at 2.95 eV, in green at 2.75 eV and in magenta at 2.64 eV.

An analogous investigation was performed on a 0.1% Ge-doped sample, observing the same spectral features, as displayed in Fig. 2a and b, reporting the spectra at different pump-probe delays and the kinetics at different probing energies, respectively. The strong noise is justified by the lower concentration of GLPC in this sample.

At this low Ge-content, the TA spectra exhibit a much well-resolved ESA band peaked at ~ 2.7 eV, which is in excellent agreement with the S_1_ → S_2_ expected transition and does not display the same important broadening observed in the high Ge-content sample. In addition, another major difference from the 6% Ge-doped sample, is the clear evidence of the change of concavity due to the tail of the PL band at 4.2 eV from the earliest acquired spectra.

**Figure 3:**
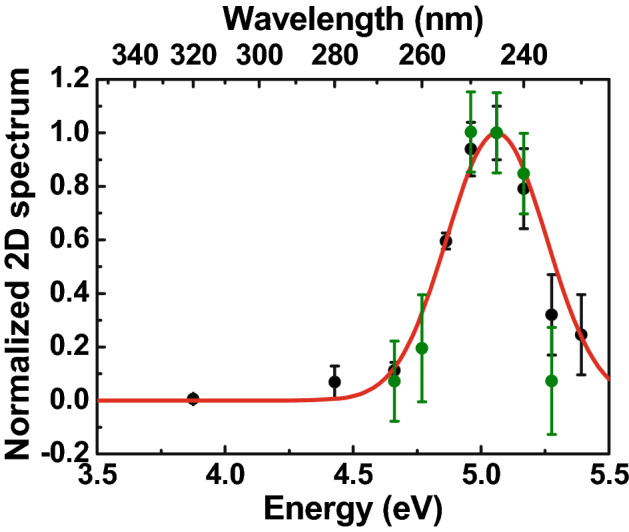
The normalized TA probed at 2.8 eV and at 5 ps of pump-probe delay as function of the exciting energy, overlapped on the normalized calculated GLPC absorption band (red line): black dots are related to the 6% Ge-doped sample and the green ones to the 0.1% Ge-doped sample.

The robustness of the considerations made above relies on the fact that we are exciting linearly and selectively the GLPC. To verify the signal’s linearity with the pump pulse energy, we have performed TA experiments varying the laser pulse energy. Figure S2 in supplementary materials reports the TA values, probed at 2.8 eV at a pump-probe delay of 5 ps in the 6% Ge-doped sample, as a function of energy per pulse; the results clearly show the linear dependence of the TA signal on the energy per pulse. Furthermore, we have performed TA experiments at different pump energies, following the GLPC’s absorption band, exciting from ~ 3.9 to ~ 5.4 eV for both samples. Figure 3 compares the calculated GLPC absorption band, based on the knowledge of its peak position and FWHM^3,21,22^ and the TA signal intensity probed at 2.8 eV at 5 ps of pump-probe delay, as a function of the exciting photon energy. The curves are normalized to their peak values and the TA levels are corrected for the laser fluence. The good agreement between the different data sets demonstrates that in our TA experiments we are selectively exciting the GLPC and then are following its photocycle.

## Discussion

All performed measurements give us the possibility to describe in detail the GLPC photocycle upon UV excitation. In particular, we have observed: (1) in the 0.1% Ge-doped sample an ESA involving the S_1_ → S_2_ electronic transition and the tail of the emission band peaking at 4.2 eV, which is instantaneously photoexcited by the femtosecond laser pulses; (2) in the 6% Ge-doped SiO_2_ sample a very large ESA band, which covers most of the investigated spectral range, suggesting an electronic transition which entails also the conduction band, and the appearance of the SE several picoseconds after the pump pulse.

**Figure 4:**
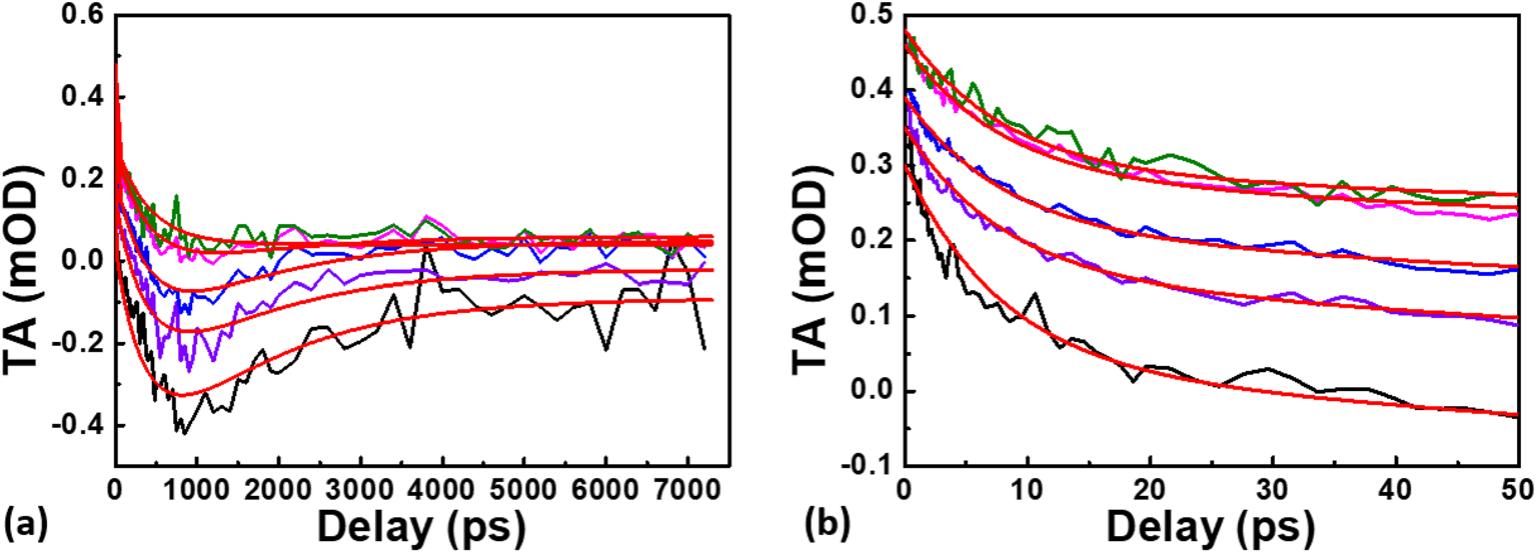
Global fitting of the different kinetics related to the GLPC’s relaxation under 5.1 eV pump excitation: in black at 3.35 eV, in purple at 3.10 eV, in blue at 2.95 eV, in magenta at 2.75 eV and in olive at 2.60 eV. The red line represents the best fitting function for each probing energy. (**a**) kinetics in the whole pump-probe delay investigated window, (**b**) zoom up to 50 ps.

To have a complete picture of the relaxation processes upon an excitation within the 5.1 eV peaked band, we have performed a fitting routine analogous to the procedure exposed in^25^ on the kinetics reported in Fig. 1c, based on global parameters used to describe the different decay components. The best-fit curves, measured on the 6% Ge-doped sample, are reported in Fig. 4, together with the TA kinetics recorded at different probing energies. The best-fit parameters are listed in Table S1 of supplementary material. The result of this fitting procedure is given by four different decay times: ~ 8 ps, ~ 400 ps, ~ 1500 ps and the last one with a timescale much longer than the one probed in the present experimental conditions. Based on the previous discussion, the decay described by the third component (1–2 ns at room temperature) can be ascribed to the combined S_1_ → S_0_ and S_1_ → T_1_ processes, which both depopulate the S_1_ singlet state at long time delays. In order to provide an interpretation of the additional two decay kinetics of 8 ps and 400 ps, we first discuss the simpler case of GLPCs in the low Ge-doped sample.

**Figure 5:**
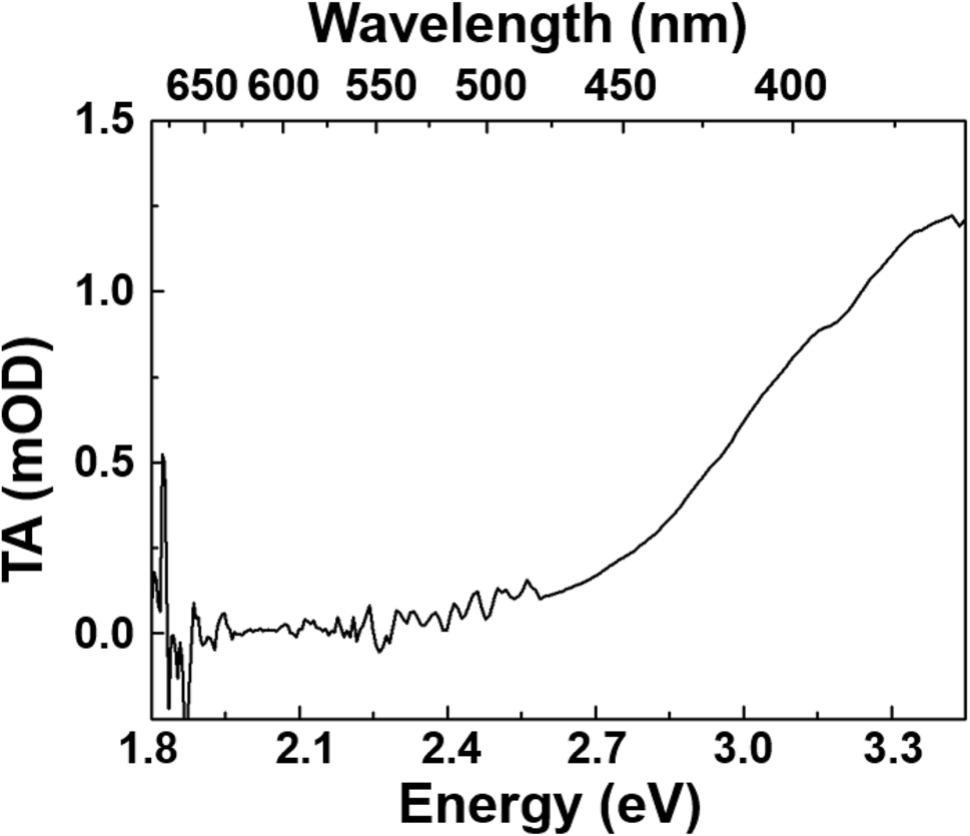
TA spectra measured at negative times in the 6% doped Ge sample: the positive signal is a fingerprint of the T_1_ → T_2_ transition.

**Figure 6:**
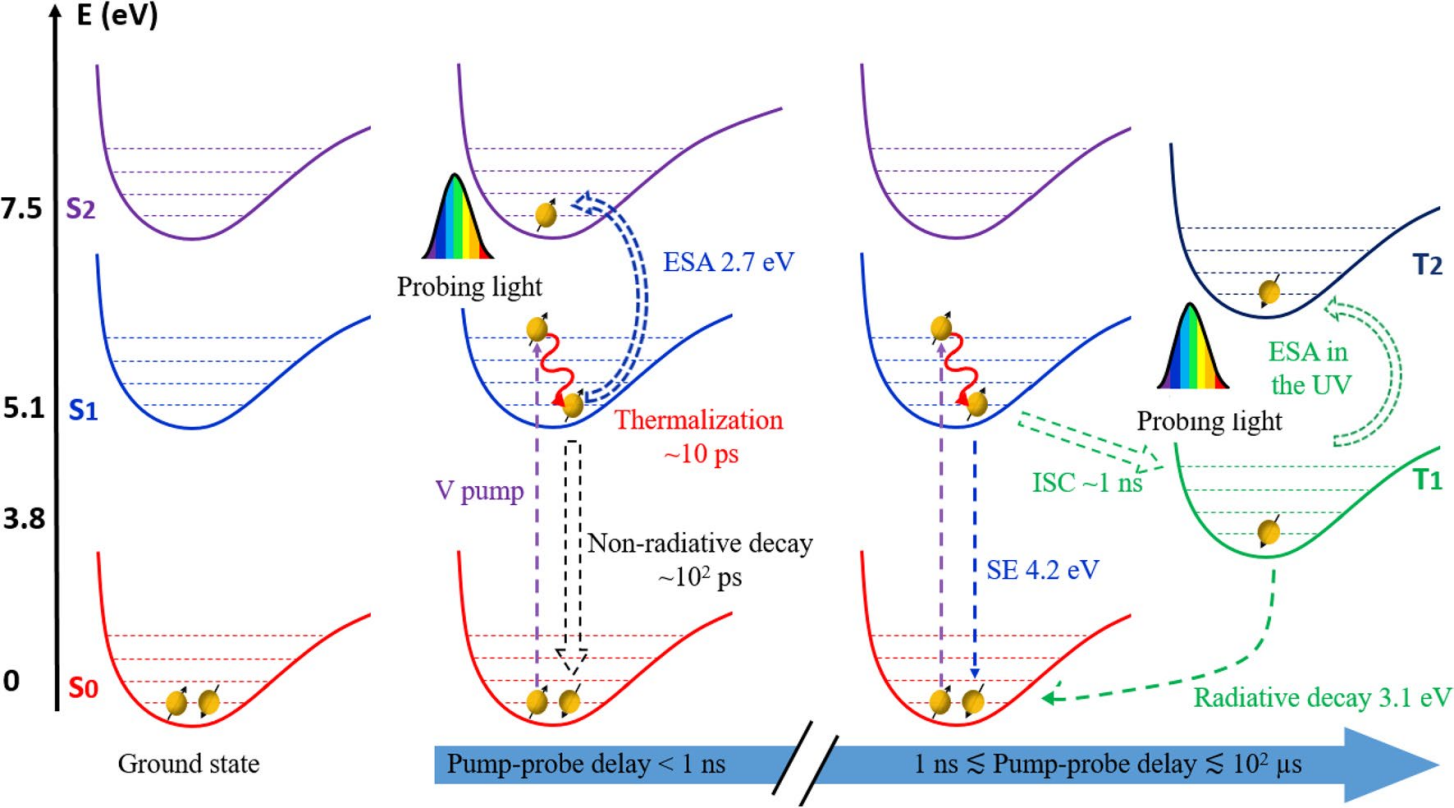
GLPC's photocycle in low Ge-content sample: the yellow spheres represent the electrons occupying the electronic levels with the corresponding spin states (black arrow up and down), dashed arrows indicate the different transitions characterizing the GLPC’s excitation/relaxation dynamics, purple and blue arrows are related to the incoming pump and probe pulse, respectively. In the figure are pictured respectively the GLPC in the ground states, the processes for pump-probes delays shorter than 1 ns and the dynamics between 1 ns and ~ 10^2^ µs leading the system newly to the ground states.

Upon UV excitation resonant with the S_1_ level, the subsequent dynamics can be interpreted as: (1) the faster decay process revealed by the experimental data (timescale ~ 8 ps) corresponds to a vibrational cooling of the populated S_1_ level, transferring the excess energy directly to the matrix, as already proposed in^32^. (2) Once the S_1_ level is thermalized, non-radiative depopulation takes place between the two singlet S_1_ → S_0_ states, justifying the decrease of the S_1_ → S_2_ ESA signal ISC and SE becomes significant. These two decay processes are observed in all the kinetics reported in Fig. 4. (3) At longer pump-probe delays, the most relevant depopulation channels of the S_1_ level, as already aforementioned, are the radiative S_1_ → S_0_ transition and the ISC between S_1_ and T_1_. It is worth to note that this contribution is present from the earliest acquired spectra in the 0.1% Ge-doped sample. (4) It is also possible to argue that the constant component (timescale much longer than the one observed in this experiment) is linked to the T_1_ state. Indeed, a few nanoseconds after the excitation, the S_1_ level should be depopulated because of the different relaxation processes already described (non-radiative and radiative S_1_ → S_0_ as well as the ISC S_1_ → T_1_). For longer delays, the only electronic level able to absorb the probe light is the T_1_ level, populated by the ISC in the nanosecond timescale^21,24^. A possible evidence of such transition is provided by pump-probe measurements with negative delays: non-zero TA signal for negative delays means that the sample is still excited in a time window comparable with the inverse of the laser repetition rate, in our case ~ 16 µs (1/60 kHz). Figure 5 reports the raw TA spectrum (without any background subtraction, as done as for the spectra in Fig. 1) measured at about − 10 ps of pump-probe delay. This hypothesis, in accordance with the long lifetime of T_1_ (~ 110 µs), suggests that the T_1_ → T_2_ absorption band is located in the UV^24^, making possible the observation of its tail. In Fig. 6 we show a simplified model of the described GLPC excitation-relaxation dynamics in the low Ge-content sample, following the relaxation mechanisms described by the previous (1)–(4) key steps.

The 6% doped Ge sample behavior needs a more complex explanation in the time window within ~ 1 ns of pump-probe delay. The high doping level strongly influences the energy bandgap, leading the GLPC to experience a different energy landscape. Indeed, the first spectra just after the pump pulse present some differences with respect to the low Ge-content sample, highlighting different dynamics at the earliest stages of the defect photocycle. Our experimental results indicate that the sample is characterized by a lower bandgap, compared to pure silica material, and the S_1_ level highly-excited vibrational states are degenerated to the conduction band. Although it is possible, in first approximation, to consider the low Ge doped sample’s electronic structure well described by a Jablonsky diagram, the interplay between S_1_ localized level and those related to conduction band, present in 6% Ge doped sample, introduces new and unexpected features. In particular, it is possible to conclude that a high Ge content in the matrix introduces a dynamic, in a timescale shorter than the nanosecond, in the defect relaxation. Indeed, upon the UV pump pulse, the electron is excited from the S_0_ ground state to a hot S_1_ state^33^, which is degenerated with the states belonging to the CB, leading to a broadening of the TA absorption band. Furthermore, the absence of the change of concavity due to the tail of the PL band at 4.2 eV, suggests that such a hot S_1_ level, mixed with the conduction band of highly Ge-doped silica, has a negligible electronic coupling with the S_0_ state, making it incapable of efficient fluorescence. For pump-probe delays longer than the electron cooling in the S_1_ level, the system reached a cold S_1_ state which is essentially identical to the one of low Ge content. At this time, an observable SE appears and the system hereafter experiences analogous excitation-relaxation dynamics as reported in Fig. 6.

## Conclusion

In the present report, we have investigated the excitation-relaxation dynamics in highly- and weakly-germanium doped amorphous silica samples with an unprecedented time resolution, highlighting the different relaxation processes in the GLPC defect. The experimental approach allows us identifying unambiguously the GLPC transient absorption response upon UV resonant excitation (5.1 eV), in linear absorption conditions and as a function of the exciting wavelength, probing in the UV–visible range. As a result, it was possible to provide a full explanation of its photocycle, fundamental to explain the characteristic relaxation mechanisms of such molecular-like systems in solid state. Moreover, the possibility to compare samples with different Ge-contents enable us to clarify the influence of the energy landscape in the relaxation dynamics of such systems. Indeed, the weakly doped sample could be described as a Jablonsky diagram characterized by typical defects electronic transition. Otherwise, the highly doped sample response underlines the interplay between the electronic transitions localized on the defect, with those related to bandgap transitions. This study highlights the Ge concentration role at the early steps of the different mechanisms (processes) and especially on the relaxation channels when approaching to short dynamics.

## Supplementary Information

Supplementary Information.

## Data Availability

The data that support the findings of this study are available from the corresponding author, V.D.M., upon reasonable request.
