# Supplementary material for: Photocycle of point defects in highly- and weakly-germanium doped silica revealed by transient absorption measurements with femtosecond tunable pump
Source: Sci Rep. 2022 Jun 2;12:9223. doi: 10.1038/s41598-022-13156-7 (PMC9163034; doi:10.1038/s41598-022-13156-7)
Supplement: Supplementary file 1 — Supplementary Information. [file 41598_2022_13156_MOESM1_ESM.docx]

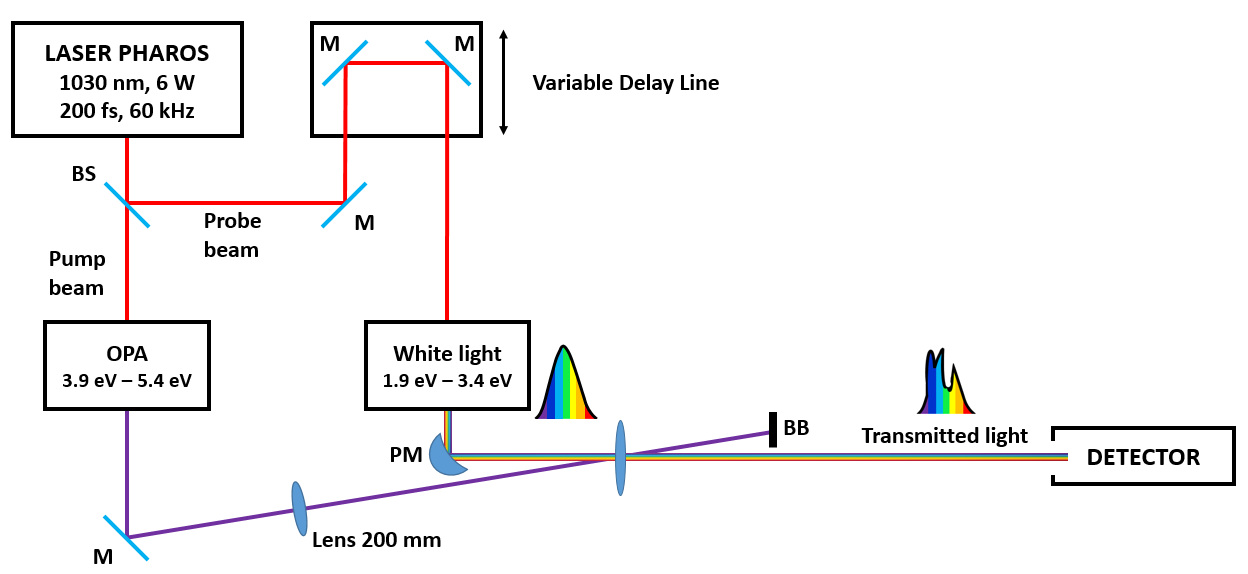


**Figure S1.** Experimental Setup used for the pump/probe measurements. BS is for beam splitter, M for mirror, PM for parabolic mirror and BB for beam blocker.

**Figure S2.** TA measured at 2.8 eV with 5 ps pump-probe delay as a function of the laser pulse energy. The red line is a guide for the reader highlighting the linear dependence.

**Table S1.** Best fit parameters of the different kinetics as a function of the pump-probe delay.

|  | $\mathbf{3.35}\mathbf{eV}$ | $\mathbf{3.10}\mathbf{eV}$ | $\mathbf{2.95}\mathbf{eV}$ | $\mathbf{2.75}\mathbf{eV}$ | $\mathbf{2.60}\mathbf{eV}$ |
| --- | --- | --- | --- | --- | --- |
| $\mathbf{A}_{\mathbf{1}}\boldsymbol{(\mu OD)}$ | $0.23$ | $0.19$ | $0.18$ | $0.21$ | $0.20$ |
| $\boldsymbol{\tau}_{\mathbf{1}}\mathbf{(ps)}$ | $8$ | $8$ | $8$ | $8$ | $8$ |
| $\mathbf{A}_{\mathbf{2}}$ $\boldsymbol{(\mu OD)}$ | $0.71$ | $0.56$ | $0.48$ | $0.23$ | $0.23$ |
| $\boldsymbol{\tau}_{\mathbf{2}}\mathbf{(ps)}$ | $400$ | $400$ | $400$ | $400$ | $400$ |
| $\mathbf{A}_{\mathbf{3}}$ $\boldsymbol{(\mu OD)}$ | $-0.57$ | $-0.39$ | $-0.32$ |  |  |
| $\boldsymbol{\tau}_{\mathbf{3}}\mathbf{(ps)}$ | $1460$ | $1460$ | $1460$ |  |  |
| $\boldsymbol{C (\mu OD)}$ | $-0.09$ | $-0.02$ | $0.05$ | $0.03$ | $0.04$ |
